# A fluorescence and UV/vis absorption dual-signaling probe with aggregation-induced emission characteristics for specific detection of cysteine

**DOI:** 10.1039/c8ra03756f

**Published:** 2018-07-05

**Authors:** Ruru Li, Xiaoyu Huang, Guolin Lu, Chun Feng

**Affiliations:** Key Laboratory of Synthetic and Self-Assembly Chemistry for Organic Functional Molecules, Center for Excellence in Molecular Synthesis, Shanghai Institute of Organic Chemistry, University of Chinese Academy of Sciences, Chinese Academy of Sciences 345 Lingling Road Shanghai 200032 People's Republic of China cfeng@sioc.ac.cn +86-21-64166128 +86-21-54925520; State Key Laboratory of Molecular Engineering of Polymers, Department of Macromolecular Science, Fudan University 220 Handan Road Shanghai 200433 People's Republic of China; School of Physical Science & Technology, ShanghaiTech University 100 Haike Road Shanghai 201210 People's Republic of China xyhuang@mail.sioc.ac.cn +86-21-64166128 +86-21- 54925310

## Abstract

Biological thiols with similar structures, such as glutathione (GSH), *N*-acetyl-l-cysteine (NAC), homocysteine (Hcy) and cysteine (Cys), play important roles in human physiology and are associated with different diseases. Thus, the discrimination of these thiols is a great necessity for various biochemical investigations and the diagnosis of related diseases. Herein, we present a new dual-signaling probe consisting of a typical aggregation induced emission fluorogen of a tetraphenylethylene group and 2,4-dinitrobenzenesulfonyl moiety. The probe can be used to selectively and quantitatively detect Cys over a variety of bio-species, including GSH, NAC and Hcy, from both UV/vis absorption and fluorescence channels. The mechanism study showed that the fluorescence and UV/vis absorption were turned on as the probe undergoes displacement of the 2,4-dinitrobenzenesulfonyl group with Cys, where the UV/vis and fluorescence signals originate from the dinitrophenyl-containing compounds and aggregates of TPE-OH, respectively. In addition, the discrimination of Cys was achieved by more rapid intramolecular displacement of sulfur with the amino group of Cys than NAC, Hcy and GSH. Moreover, the probe shows ignorable cytotoxicity against HepG2 cells, which demonstrates the great potential of the probe in selectively detecting Cys *in vivo*.

## Abstract 2

A dual-signaling of fluorescence and UV/vis absorption modes for selective and quantitative detection of cysteine over homocysteine, *N*-acetyl-l-cysteine and glutathione is developed on the basis of aggregation-induced emission (AIE) effect.

## Graphical abstract

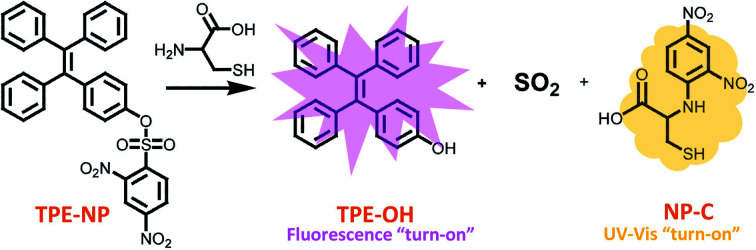

## Introduction

Biological thiols such as glutathione (GSH), *N*-acetyl-l-cysteine (NAC), homocysteine (Hcy) and cysteine (Cys) play essential roles in human physiology.^1–6^ Although these thiols have similar structure, their roles in biological processes are diverse and associated with different diseases. For instance, GSH is a pivotal indicator of the maintenance of xenobiotic metabolism, intracellular signal transduction and gene regulation, which is highly involved in the aging and the pathogenesis of many diseases.^1,2^ Since NAC was found to be a precursor of GSH in cells and a thiol antioxidant, it shows the potential to prevent cancer and other mutation-related diseases.^6^ Hcy is a risk factor for Alzheimer's and cardiovascular diseases.^3,4^ Abnormal level of Cys would lead to slow growth, skin lesions, liver damage and edema *etc*.^5^ Due to the critical role of these thiols in human health, the selective detection of these thiols is of great importance and a necessity for various biochemical investigations and the diagnosis of related diseases. However, it is challenging to selectively and quantitatively detect these thiols because of the structural similarity of Cys, Hcy, NAC and GSH.^7–10^

In the past few years, some fluorescent and colorimetric probes have been developed to selectively detection Cys in terms of high sensitivity and simplicity of operation.^10,11^ However, most of these probes send out only one signal based on the same chemo-sensor molecule,^12–14^ which may be influenced by possible variations in the sample environment, especially in biological surroundings. In order to improve the accuracy of the results, the utilization of dual signaling probes is one of effective alternatives, which allows mutual correction on the same target analyte, yet through different transduction channels.^15–20^ However, dual signaling probes for Cys are still very limited so far.^10,11,21^

In 2001, Ben Zhong Tang reported an abnormal phenomenon of aggregation induced emission (AIE) of silole, which shows nonfluorescence in solutions but strong emission once aggregated.^22–24^ By taking advantage of attractive AIE phenomenon, a large number of intelligent AIE fluorogens susceptible to external stimuli, such as bio-species, mechanical force, temperature change, pH variation, toxic vapor, and photonic irradiation have been explored and developed.^25^ Tetraphenylethylene (TPE) is a typical AIE fluorogen,^26–30^ which has been widely used for the construction of various fluorescent sensors due to its significant increase in fluorescence upon aggregation, low cytotoxicity and ease in synthesis and modification.^31–37^ Just recently, Wang and co-workers reported a new TPE-based AIE probe bearing a 2,4-dinitrobenzenesulfonyl pendant, which is able to detect Cys selectively over Hcy and GSH in PBS.^38^ In this work, they found that fluorescence would significantly increase upon the substitution reaction between the probe with Cys, which would result in the leave of fluorescent quencher of 2,4-dinitrobenzenesulfonyl group from TPE moiety. Although previous works showed that some 2,4-dinitrobenzenesulfonyl-based probes were not able to discriminate Cys from GSH or Hcy,^39,40^ this probe demonstrated a strong selectivity to Cys over Hcy and GSH. All these results showed that the whole structure of probes, besides 2,4-dinitrobenzenesulfonyl itself, also affect the reactivity of substitution reaction toward thiol compounds, especially the reaction kinetics.^38–40^

**Scheme 1:**
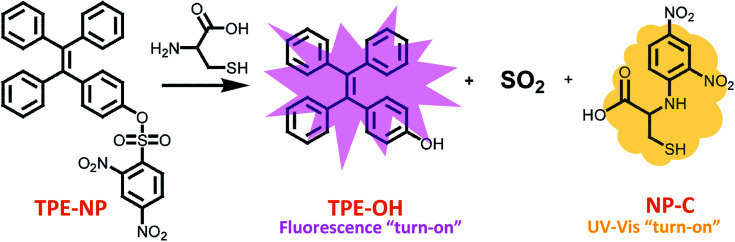
Structures of TPE-NP sensor and its products upon treating with Cys.

Herein, a TPE-based dual signaling probe was synthesized by introducing a 2,4-dinitrobenzenesulfonyl group to a TPE moiety (Scheme 1).^41^ It is assumed that the 2,4-dinitrobenzenesulfonyl group would not only make the TPE-based probe more soluble in aqueous media, but also quench the possible fluorescence of TPE through photo-induced electron transfer (PET). Thus, the replacement of 2,4-dinitrobenzenesulfonyl group by Cys would not only significantly increase the fluorescent of TPE by the combination of AIE and PET effect, but also enhance the UV/vis absorption due to the producing of dinitrobenzene-based compound. In addition, it is hypothesized that the discrimination of Cys could be achieved by more rapid intramolecular displacement of sulfur with amino group of Cys than NAC, Hcy and GSH. Therefore, the probe would be able to selectively detect Cys over NAC, Hcy and GSH through the combination of fluorescence and UV/vis absorption from two separated transduction species. To test this hypothesis, the performance of TPE-NP for selectively detecting Cys over other various bio-species, including NAC, Hcy and GSH were examined by Fluorescent and UV/vis absorption spectroscopy and the mechanism behind was also investigated by the combination of HPLC-MS and ^1^H NMR. It should be pointed out that although the strategy is similar to that of previous report,^38^ where only fluorescent channel was employed to detect Cys, a dual signaling approach was develop in the current work, which will allows to mutual correction on the same target analyte to get more reliable results. In addition, the reaction mechanism for selectively detecting Cys was systematically investigated in the current work, which is of great benefit to design highly selective and sensitive dual signaling probes for detecting biological thiols.

## Experimental

### Materials

Tetrahydrofuran (THF, Aldrich, 99%) was dried over CaH_2_ and distilled from sodium and benzophenone under N_2_ prior to use. 4-Hydroxybenzophenone (Acros, 99%), 2,4-dinitrobenzenesulfonyl chloride (Alfa Aesar, 98%), benzophenone (Acros, 99%), l-cysteine (Cys, Acros, 99%), l-glutathione (GSH, Acros, 99%) and dl-homocysteine (Hcy, TCI, 90%) were used as received without further purification. Cell Counting Kit-8 (CCK-8, Dojindo, Japan) and DMEM medium (GIBCO/Invitrogen, U.S.A). Other reagents not specially mentioned were purchased from Aladdin and used as received without further purification.

### Instrumentation

All ^1^H and ^13^C NMR analyses were performed on a JEOL resonance ECZ 400S (400 MHz) in DMSO-*d*_6_, tetramethylsilane was used as internal standard. Electron impact ionization mass spectrometry (EI-MS) was performed by an Agilent 5937N system. FT-IR spectra were recorded on a Nicolet AVATAR-360 spectrophotometer with a 4 cm^−1^ resolution. UV/vis absorption spectra were recorded on a Hitachi U-2910 spectrophotometer. Fluorescence spectra were measured by a Hitachi F-2700 fluorescence spectrophotometer with a band width of 2.5/5.0 nm. UV/vis and fluorescence measurements were performed under room temperature.

### Synthesis of 4-(1,2,2-triphenylvinyl)phenol (TPE-OH)

Benzophenone (7.26 g, 39.85 mmol), 4-hydroxybenzophenone (7.90 g, 39.85 mmol), zinc powder (11.46 g, 175.34 mmol) and THF (200 mL) were added to a three-neck flask equipped with a magnetic stirrer under N_2_. The mixture was cooled to 0 °C followed by adding TiCl_4_ (16.63 g, 87.67 mmol) slowly *via* a constant pressure drop funnel while the temperature was kept under 0 °C. The suspending mixture was warmed to room temperature, stirred for 0.5 h and then heated at reflux for another 15 h. The mixture was cooled to room temperature and charged with HCl (320 mL, 1 M). The mixture was extracted by CH_2_Cl_2_ and the organic layer was dried over MySO_4_ and concentrated. The crude product was purified by silica column chromatography (eluent: *n*-hexane/ethyl acetate, v/v = 4/1) to give 4-(1,2,2-triphenylvinyl)phenol (TPE-OH) (9.02 g, 65%) as a white powder.

^1^H NMR: *δ* (ppm): 9.37 (s, 1H, OH), 7.10 (m, 9H, ArH), 6.97 (t, 6H, ArH), 6.75 (d, 2H, ArH), 6.48 (d, 2H, ArH).

### Synthesis of 4-(1,2,2-triphenylvinyl)phenyl 2,4-dinitrobenzenesulfonate (TPE-NP)

TPE-OH (4.50 g, 12.90 mmol), 2,4-dinitrobenzenesulfonyl chloride (6.88 g, 25.80 mmol), CH_2_Cl_2_ (200 mL) and Et_3_N (2.61 g, 25.80 mmol) were added to a three-neck flask equipped with a magnetic stirrer under N_2_. The mixture was stirred at room temperature for 14 h. The solution was diluted with CH_2_Cl_2_ (200 mL), washed with 1 M HCl and brine (200 mL), and dried over MySO_4_. The solvent was removed by rotary evaporation, and the crude product was purified by silica gel chromatography (eluent: *n*-hexane/ethyl acetate, v/v = 5/1) to give 4-(1,2,2-triphenylvinyl)phenyl 2,4-dinitrobenzenesulfonate (TPE-NP) (5.99 g, 80%) as a yellow powder.

^1^H NMR: *δ* (ppm): 9.08 (s, 1H, ArH), 8.59 (d, 1H, ArH), 8.10 (d, 1H, ArH), 7.11 (m, 9H, ArH), 6.94 (m, 10H, ArH).

^13^C NMR: *δ* (ppm): 121.16, 121.57, 126.85, 127.42, 128.03, 130.68, 132.72, 133.62, 139.09, 114.83, 142.56, 142.85, 143.47, 146.85, 148.28, 151.47.

MS (ESI): calc. for C_32_H_22_O_7_N_2_S ([M + 23]^+^): 601.11; found: 601.11.

### Synthesis of 4-phenyl 2,4-dinitrobenzenesulfonate (phenol-NP)

Phenol (1.00 g, 10.60 mmol), 2,4-dinitrobenzenesulfonyl chloride (5.66 g, 21.23 mmol), CH_2_Cl_2_ (50 mL) and Et_3_N (2.15 g, 21.25 mmol) were added to a three-neck flask equipped with a magnetic stirrer under N_2_. The mixture was stirred at room temperature for 12 h. The solution was diluted with CH_2_Cl_2_ (50 mL), washed with 1 M HCl and brine (50 mL), and dried over MySO_4_. The solvent was removed by rotary evaporation, and the crude product was purified by silica gel chromatography (eluent: *n*-hexane/CH_2_Cl_2_, v/v = 3/1) to give 4-phenyl 2,4-dinitrobenzenesulfonate (phenol-NP) (2.30 g, 67%) as a white powder.

^1^H NMR: *δ* (ppm): 9.11 (s, 1H, ArH), 8.60 (d, 1H, ArH), 8.21 (d, 1H, ArH), 7.12 (m, 3H, ArH), 6.93 (m, 2H, ArH).

### Cytotoxicity assay *in vitro* against HepG2 cells

The cytotoxicity of the samples were assessed against HepG2 cells with the CCK-8 assay. In brief, the HepG2 cells were respectively incubated with the TPE-NP with different concentrations for 24 h. After that, the cells were resuspended in fresh medium and treated with DMEM solution. Furthermore, the control group was only treated with physiological saline. The evaluation of cytotoxicity was determined by cell viability which was calculated on the basis of the absorbance of control group and the absorbance of treated group.

## Results and discussion

### Synthesis of probe TPE-NP

**Fig. 1:**
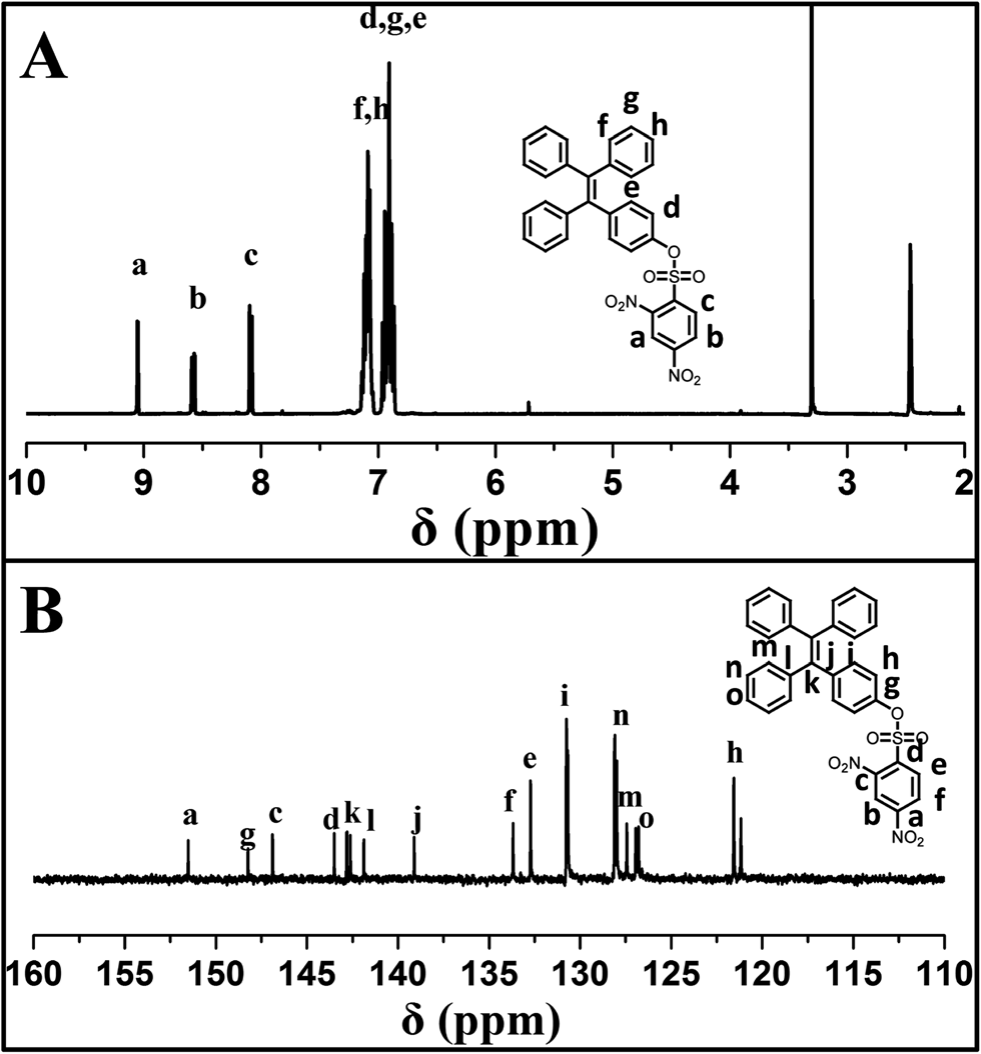
(A) ^1^H NMR and (B) ^13^C NMR spectra of TPE-NP in DMSO-*d*_6_.

4-(1,2,2-triphenylvinyl)phenol (TPE-OH) was prepared firstly from benzophenone and 4-hydroxybenzophenone according to previous report. Then, the sensor of 4-(1,2,2-triphenylvinyl)phenyl 2,4-dinitrobenzenesulfonate (TPE-NP) was readily synthesized by the reaction of TPE-OH with 2,4-dinitrobenzenesulfonyl chloride. The structure of TPE-NP sensor was well characterized by ^1^H NMR, ^13^C NMR, FT-IR and MS. Fig. 1A shows ^1^H NMR spectrum of TPE-NP, which exhibits the resonance signals of 2,4-dinitrophenyl group (peak “a”, “b” and “c”) at 9.08, 8.60 and 8.12 ppm and TPE moiety at 7.11 and 6.94 ppm. The ^13^C NMR spectrum of TPE-NP demonstrates the expected resonance signals of TPE and 2,4-dinitrophenyl groups (Fig. 1B). In addition, the ESI-MS result (578.11 g mol^−1^) was also consistent with the theoretical value (578.11 g mol^−1^). All these results confirmed the successful synthesis of target sensor TPE-NP.

### Selectively and quantitatively detection of Cys by TPE-NP

**Fig. 2:**
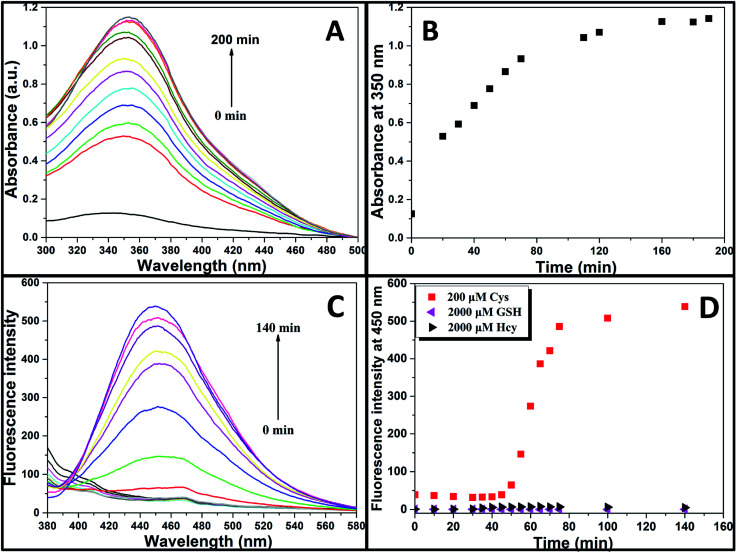
(A) UV/vis spectra, (B) dependence of absorbance intensity at 350 nm with time, and (C) fluorescent spectra of TPE-NP solution (240 μM in PBS/DMSO (v/v = 8/2), pH = 7.4) in the presence of Cys (200 μM). (D) The time course of the fluorescence intensity at 450 nm of TPE-NP solution (240 μM in PBS/DMSO (v/v = 8/2), pH = 7.4) in the presence of 200 μM Cys and 2000 μM of Hcy and GSH.

Aqueous solution of TPE-NP (240 μM in PBS/DMSO (v/v = 8/2), pH = 7.4) is colorless because of the absence of absorption features in the visible region (Fig. 2A). The solution exhibits no fluorescence, probably due to relatively high solubility of TPE-NP in the media and photo-induced electron transfer between tetraphenylethene group and dinitrophenyl moiety (Fig. 2C).^15,42,43^ Upon the addition of Cys (200 μM), the colorless solution became bright yellow. This observation showed that TPE-NP could be a “naked-eye” probe for Cys. Meanwhile, the absorption band at around 350 nm increased steadily over time and reached plateau after about 2 h (Fig. 2B). Upon the addition of Cys, a new emission peak located at 450 nm emerged and a dramatic fluorescence enhancement was observed in Fig. 2C. The time-dependent fluorescence response was also detected, which showed that the fluorescence intensity at 450 nm almost does not change before 50 min but then increased sharply to about more than ten times of the weak background after 80 min (Fig. 2D). Although the steady increase of absorption at 350 nm indicated that the displacement reaction occurred continually upon the addition Cys, the product of TPE-containing moiety did not aggregate until its concentration is higher than its critical aggregation concentration. This might be the reason why the fluorescent intensity just increased obviously after around 50 min upon the treatment with Cys.

**Fig. 3:**
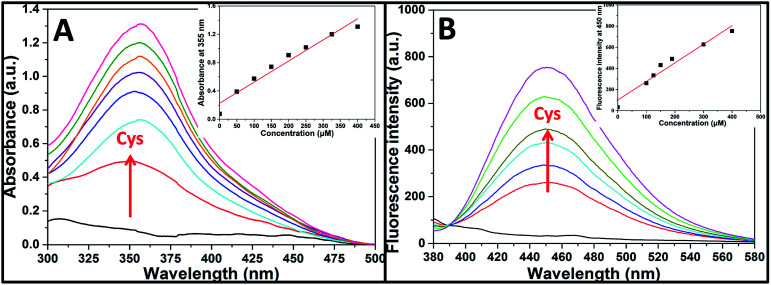
(A) UV/vis and (B) fluorescence spectra of TPE-NP solution (240 μM in PBS/DMSO (v/v = 8/2), pH = 7.4) as a function of Cys concentration. The insets are the absorbance at 350 nm and the fluorescence intensity at 450 nm as a function of Cys concentration, respectively.

We then incubated the solution with various concentrations of Cys (20–400 μM) for 2 h. One can notice that both UV/vis absorption and fluorescent intensity of TPE-NP solution increase with the increasing in the concentration of Cys (Fig. 3A and B). We plotted the absorbance intensity at 350 nm and fluorescence intensity at 450 nm of TPE-NP solution as a function of the concentration of Cys. Both absorbance and fluorescence intensities are linearly proportional to the concentration of Cys (insets of Fig. 3A and B), resulting the detection limit of Cys in PBS low to 0.21 μM (S/N = 3) from fluorescent channel. In previous reports on the detection of Cys by fluorescent sensors, the detection limit is in the range from 0.121 μM to 1 μM.^44–46^ Especially, the detection limit of Cys for a TPE-based probe reported by Wang and co-workers is about 0.18 μM, close to the value of TPE-NP.^38^ All these results show that Cys can be quantitatively detected on the basis of the change in UV/vis absorption and fluorescence intensities.

**Fig. 4:**
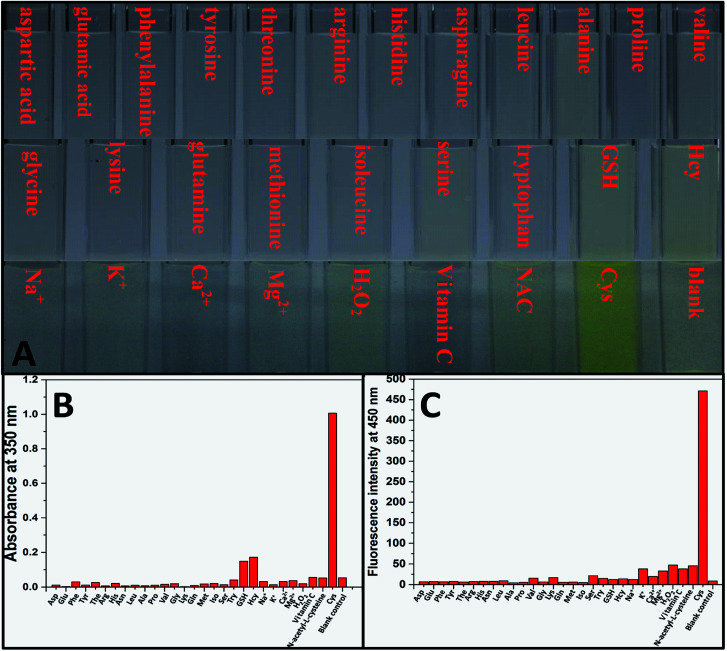
(A) Photographs, (B) UV/vis absorbance at 350 nm and (C) fluorescence intensity at 450 nm of TPE-NP solution (240 μM in PBS/DMSO (v/v = 8/2), pH = 7.4) upon incubation with 200 μM of various bio-species, including amino acids (aspartic acid, glutamic acid, phenylalanine, tyrosine, threonine, arginine, histidine, asparagine, leucine, alanine, proline, valine, glycine, lysine, glutamine, methionine, isoleucine, serine, tryptophan, GSH and Hcy), metal ions (Na^+^, K^+^, Ca^2+^ and Mg^2+^), H_2_O_2_, vitamin C and NAC for 2 h, respectively.

We then test selectivity of TPE-NP by examining UV/vis absorption and fluorescence responses of TPE-NP toward Cys and a variety of bio-species, including amino acids (aspartic acid, glutamic acid, phenylalanine, tyrosine, threonine, arginine, histidine, asparagine, leucine, alanine, proline, valine, glycine, lysine, glutamine, methionine, isoleucine, serine and tryptophan), as well as common biological metal ions (Na^+^, K^+^, Ca^2+^ and Mg^2+^), reactive oxygen species (H_2_O_2_), vitamin C, GSH, NAC and Hcy. It was found that these common bio-species did not cause significant changes in color (Fig. 4A), UV/vis absorption (Fig. 4B) and fluorescence (Fig. 4C) of solution except Cys. Especially, GSH, Hcy and NAC, three thiol-containing compounds as Cys, did not cause obvious change in fluorescent emission, even the amount of Hcy and GSH are ten times of that of Cys (Fig. 2D and 4C). All these results show that TPE-NP has a high selectivity toward Cys over Hcy, NAC, GSH and other related bio-species, even if the only difference of Hcy and NAC from Cys is a methylene or an acetyl group at their residues.

### Mechanism of detecting Cys by TPE-NP

Since dinitrophenyl group is a well-known fluorescent quencher, the fluorescence of TPE-NP is probably quenched by dinitrophenyl group due to PET effect.^15,42,43^ On the basis of previous reports,^15,42,43^ we speculated that the displacement of dinitrophenyl group of TPE-NP upon the treatment with Cys gave TPE-OH and dinitrophenyl-containing compounds (Scheme 1). Thus, fluorescence emission of TPE-OH at 450 nm was turned on and the produce of dinitrophenyl-based compound resulted in the yellow color of solution (Scheme 1).

**Fig. 5:**
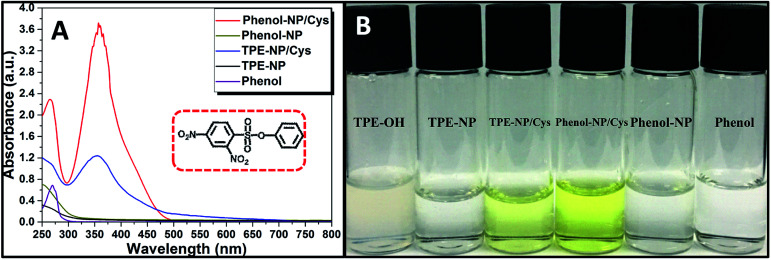
(A) UV/vis spectra of TPE-NP and phenol-NP solutions (240 μM in PBS/DMSO (v/v = 8/2), pH = 7.4) with and without the treatment with Cys and phenol. (B) Photographs of TPE-NP and phenol-NP solutions (240 μM in PBS/DMSO (v/v = 8/2), pH = 7.4) with and without treatment with Cys, TPE-OH and phenol. Inset of part (A) is the structure of phenol-NP.

In order to testify the yellow color originated from dinitrophenyl-containing compound, we synthesized a new sensor of 4-phenyl 2,4-dinitrobenzenesulfonate (phenol-NP) by the reaction between phenol, instead of TPE-OH, and 2,4-dinitrobenzenesulfonyl chloride (Fig. 5A). After the aqueous solution of phenol-NP (240 μM in PBS/DMSO (v/v = 8/2), pH = 7.4) was treated with Cys (200 μM), the colorless solution turned yellow (Fig. 5B) and the absorption with a maximum wavelength at about 350 nm obviously increased (Fig. 5A). Whereas, aqueous solutions (PBS/DMSO (v/v = 8/2), pH = 7.4) of TPE-OH and phenol is colorless (Fig. 5B). Thus, these results indicate that the fluorescence and UV/vis absorption (yellow) are originated from the aggregates of TPE-OH and dinitrophenyl-containing compounds, respectively. This means that the dual signals of UV/vis absorption and fluorescence for detecting Cys quantitatively and simultaneously based on TPE-NP probe are originated from two separated signaling molecules. Thus, the cross-referencing of the obtained results can be realized by this approach, which undoubtedly makes the results more reliable.

**Fig. 6:**
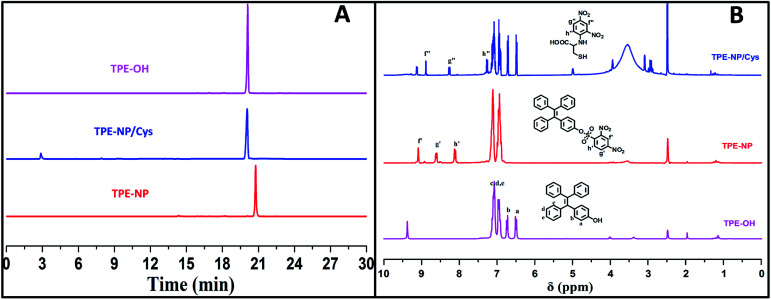
(A) HPLC curves and (B) ^1^H NMR spectra in DMSO-*d*_6_ of TPE-OH and TPE-NP before and after treating with Cys.

To verify the mechanism of the reaction of TPE-NP with Cys, the products of the reaction were analyzed by HPLC and ^1^H NMR (Fig. 6A and B). We can see that a peak at 20.1 min appears in HPLC curve (Fig. 6A) after TPE-NP was incubated with Cys for 2 h, where the retention time is the same as that of TPE-OH (20.1 min). Moreover, the molecular weight of this compound is 348.1 g mol^−1^, consistent with that of TPE-OH (348.1 g mol^−1^). One also can notice that two characteristic proton resonance signals of TPE-OH at 6.47 and 6.70 ppm and three peaks originated from the protons of dinitrophenyl group at 8.95, 8.86 and 7.23 ppm appear after the incubation (Fig. 6B). These observations show that TPE-OH is one of the products of the reaction and the up-shift of signals from dinitrophenyl moiety suggests the leave of electron withdrawing group.

**Fig. 7:**
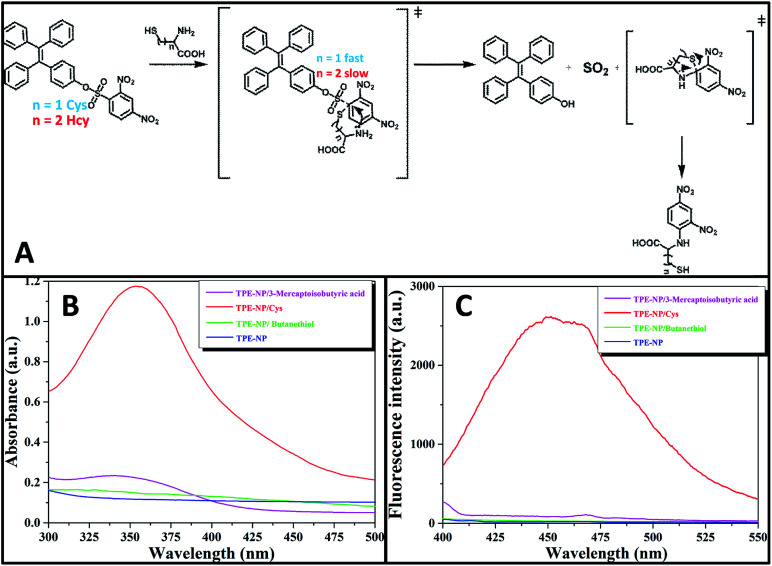
(A) Proposed mechanism for the reaction of TPE-NP with Cys and Hcy. (B) Fluorescence and (C) UV/vis spectra of TPE-NP solution (240 μM in PBS/DMSO (v/v = 8/2), pH = 7.4) in the presence of 3-mercaptoisobutyric acid, butanethiol and Cys (200 μM), *λ*_ex_ = 350 nm, respectively.

Since all of Cys, Hcy, NAC and GSH contain a thiol group, one inevitably raises the question why Cys has much higher reactivity toward TPE-NP than Hcy, NAC and GSH. On the basis of previous reports on the detection of thiols using nitrophenyl-containing sensors^15,42,43^ and the above observations, a possible mechanism is proposed as shown in Fig. 7A. At pH = 7.4, the deprotonated thiols of Cys, Hcy, NAC and GSH would serve as active nucleophile to attack the dinitrophenyl of TPE-NP for generating a sulfur-substituted intermediate (Fig. 7A). The primary amine of Cys, Hcy and GSH would further attack the dinitrophenyl to form thermodynamic five-, six- or ten-membered cyclic transition state by intramolecular cyclization, which would assist the leave of TPE-containing moiety to give TPE-OH and SO_2_. Since five-membered ring is much more favored conformation than six- and ten-membered ring, the reaction rate for Cys would be much faster than those of Hcy and GSH. To verify the importance of amino group in accelerating the displacement reaction, TPE-NP solution was incubated with butanethiol and 3-mercaptoisobutyric acid for 2 h. In comparison to Cys, both butanethiol and 3-mercaptoisobutyric acid almost did not lead to obvious change in fluorescence and UV/vis spectra (Fig. 7B and C). The low reactivity of TPE-NP toward NAC further shows the importance of amino group in accelerating the displacement reaction. All these results demonstrate that the proposed reaction process is reasonable.

### *In vitro* cytotoxicity of TPE-NP

**Fig. 8:**
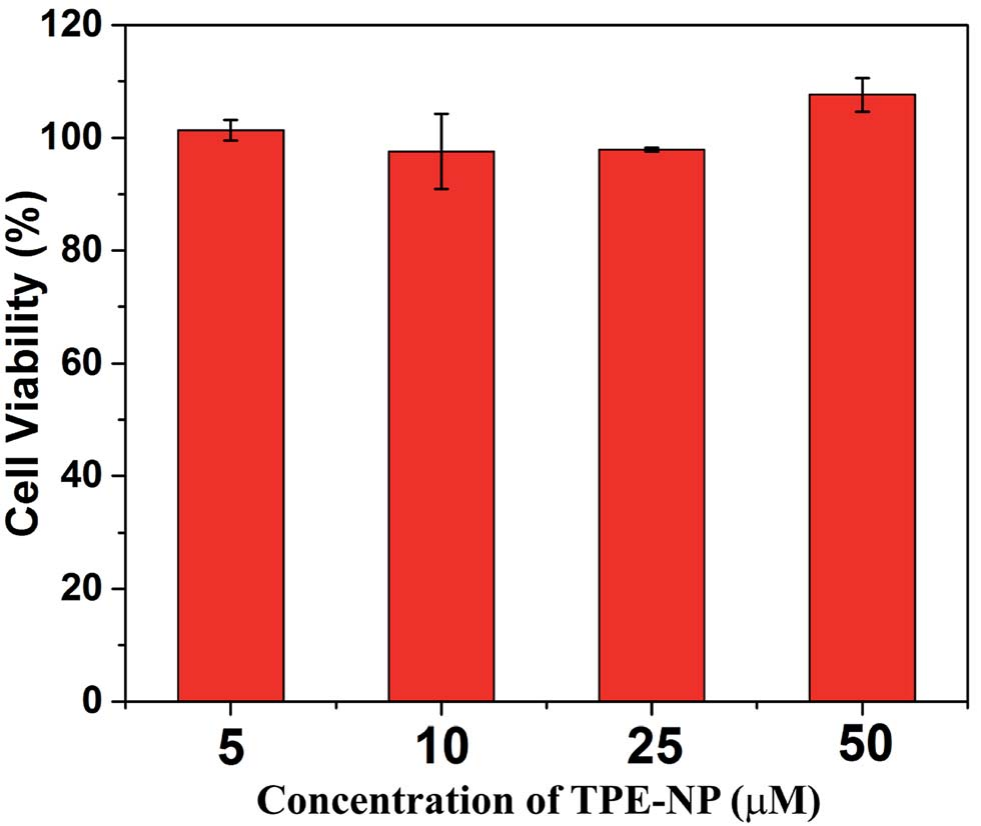
*In vitro* cytotoxicity of TPE-NP against HepG2 cells.

Finally, we performed proof-of-concept *in vitro* experiments on the examination of the *in vitro* cytotoxicity of TPE-NP against the HepG2 cells, which was evaluated by means of a standard CCK-8 cell assay. It could be seen that the cell viability of TPE-NP is higher than 95% for HepG2 cells in a range of concentrations from 5 to 50 μM, respectively. These results show that TPE-NP have ignorable cytotoxic effect on the HepG2 cells in a range of concentration (5–50 μM, 2.89–28.9 μg mL^−1^), which demonstrates the potential of TPN-NP in selectively detecting Cys *in vivo* (Fig. 8).

## Conclusions

In summary, a simple colorimetric and fluorescent dual signaling probe has been developed, and the probe can selectively and quantitatively detect Cys from other common bio-species including Hcy, NAC and GSH. The fluorescence and UV/vis absorption are originated from the aggregates of TPE-OH and dinitrophenyl- containing compound, respectively. This allows the mutual correction through different transduction channels and signaling reporters, which will make the result more reliable. It is proposed that the discrimination of Cys from Hcy, NAC and GSH is achieved through the faster displacement of sulfonate attaching to dinitrophenyl with thiolate for Cys, followed by subsequent substitution of the thiolate by the amino group of Cys. Given the low cytotoxicity of TPE-NP, the interesting reaction mechanism provides a strategy for designing highly selective and sensitive dual signaling probes for detecting Cys *in vitro* and *in vivo*.

## Conflicts of interest

There are no conflicts to declare.

## Supplementary Material
